# Africa’s Indigenous Fruit Trees: A Blessing in Decline

**DOI:** 10.1289/ehp.123-A291

**Published:** 2015-12-01

**Authors:** Rachel Cemansky

**Affiliations:** Rachel Cernansky is a freelance journalist in Denver, Colorado, covering science, health, and the environment. She has written for publications including *Yale Environment 360*, *Nature*, *Civil Eats*, and *The New York Times*.

For probably as long as people have lived in Africa, they have eaten culturally and traditionally important indigenous fruits such as baobab, desert date, black plum, and tamarind. Farmers have been able to enjoy the fruit of these plentiful wild trees without developing any knowledge of how to propagate them successfully; they haven’t needed to.

**Glossary**

**Agroforestry:** A land management system that integrates trees within other crops or livestock systems to improve the complex beneficial interactions with other organisms (bacteria, fungi, insects, birds, mammals) and so improve the ecological health of the system.

**Breeding:** Cross-pollinating plants to produce a new plant with desirable qualities from both parents. In trees this is a relatively slow and inefficient form of domestication.

**Crop diversification:** Introducing additional crops to what’s grown on a farm.

**Crop intensification:** Using various methods to enhance crop yield.

**Cropping system:** The pattern of crops grown and farming practices employed on a given field or farm. For instance, a polyculture cropping system involves growing a diversity of crops simultaneously in the same space. In conventional agriculture, cropping systems are often monocultures (growing a single crop at a time).

**Cultivar:** A plant variety that is produced by breeding.

**Cultivation:** Growing crops in a farm field.

**Cutting:** A section of shoot or root cut from a plant, which is encouraged to form a new plant by developing roots and shoots (see “Rooting”). In contrast to plants grown from seeds, cuttings remain “true” to the plant from which they are taken.

**Domestication:** Intentionally growing plants that originated in the wild, a process enhanced by selecting the best specimens and propagating them vegetatively (e.g., by taking cuttings as opposed to planting seeds).

**Grafting:** Taking sections of branch from a mature tree and fusing them onto a small seedling. The result is a new plant with the characteristics of the mature tree. “Multigrafted” trees produce a variety of fruit on the same tree, but this method is generally more of a novelty than a production system.

**Propagation:** The act of producing new plants by any method.

**Rooting:** Stimulating a cutting to develop and grow roots, typically by inserting the cutting into a humid environment with a damp, loosely packed medium such as peat moss or vermiculite.

**Smallholder:** A farmer working 2–5 hectares of land with a combination of cash crops to sell and food crops to feed and support the family.

However, the trees’ natural habitats are being lost, mainly to widespread deforestation resulting from population growth, the cutting of trees for firewood or charcoal, and in some cases industrial agriculture or other business interests. With this loss, questions arise over where the trees will grow in the future, if at all—and whether they will continue to provide the same wide range of benefits if they do survive.

**Figure:**
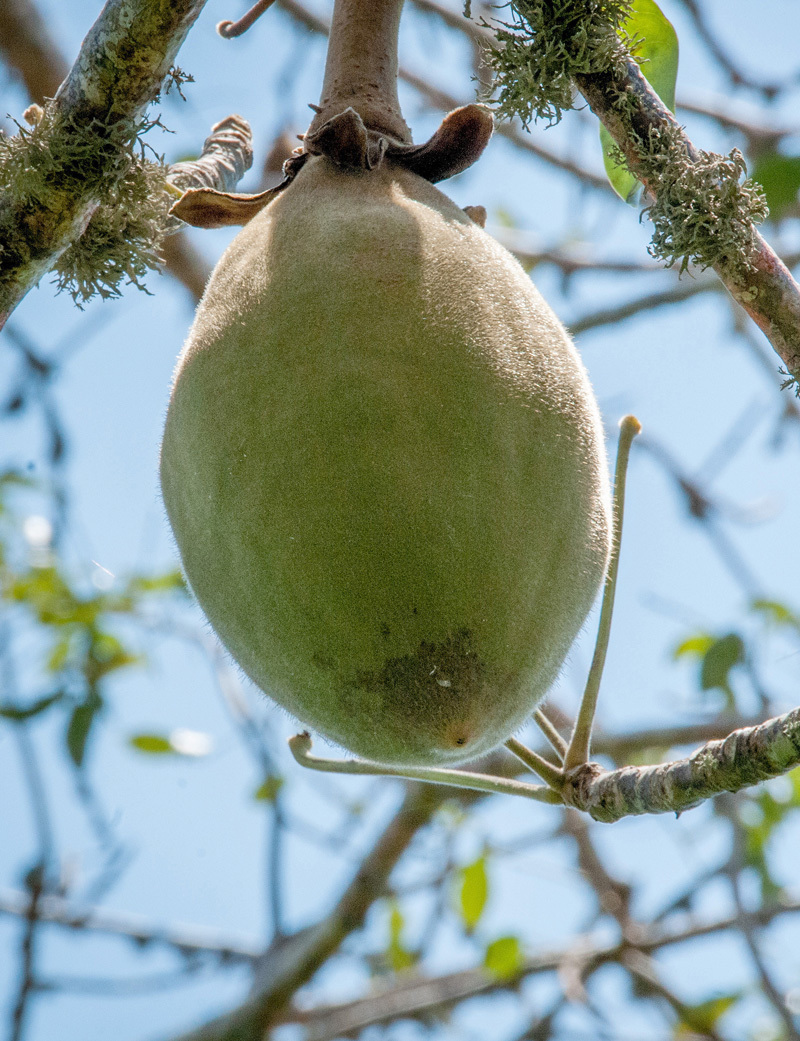
Fruits native to the African continent offer nutrients that often come up short in local diets, and they grow on trees that provide a range of ecosystem services. But deforestation is threatening Africa’s indigenous fruit trees. Now researchers and local farmers are partnering to ensure the trees have a future. © Ake Mamo/ICRAF

Trees of all kinds provide immeasurable ecosystem services, including carbon sequestration, continual replenishment of soil, and removal of pollutants from air.^1^ Wooded areas foster biodiversity, which provides benefits of its own. For instance, deforestation is associated with increased risk of malaria transmission due to an increase in mosquito breeding sites on cleared land and reduced populations of mosquito predators.^2^

What’s unique about fruit trees is their ability to provide vital nutrients that may otherwise be scarce.^3^ Indigenous fruit trees, in particular, have still another added benefit: Naturally adapted to local soils and climates, wild trees often survive environmental stresses better than introduced species.^4^

A growing number of researchers, conservationists, and plant domesticators are fighting to reverse the population declines these native fruit trees are experiencing. Across Africa and in other parts of the world, scientists are studying their nutritional and ecological benefits and how those properties could be enhanced if the wild trees are domesticated. They’re also characterizing the genetic diversity of the trees and working with growers to ensure their successful cultivation, often as new crops, with the potential to transform local agriculture.^5^

Farmers are considered a crucial part of preserving the future for indigenous fruit trees, which, in turn, are viewed as a means for improving the livelihoods of poor smallholder farmers in particular. “The future of trees is on farms,” says Ramni Jamnadass, leader of the Quality Trees Global Research Project at the Nairobi-based International Centre for Research in Agroforestry (ICRAF). The center, also known as the World Agroforestry Centre, is the driving force of much of the research on African indigenous fruits.^6^ “If we don’t invest in them now,” Jamnadass says, “we will lose many of the species, as many of them are fast disappearing.”

## A Future for Fruit Trees

Plenty of African farmers grow commercially popular fruits that are exotic to the region, such as avocadoes, oranges, mangoes, and papayas. However, relatively few intentionally grow indigenous fruits. “There are more than eight hundred fruit species in this region alone, and how many do you see in the market—three or four. So there’s huge potential that will be lost if we don’t do something,” says Jamnadass.

A little over a decade ago, the same disparity between natural variety and market representation existed for indigenous vegetables in parts of Africa. Many vegetables have made a resurgence on the market and are not only available for purchase but often in higher demand than their exotic counterparts, which in some regions were long considered more desirable.^7^

The same shift does not seem to be happening for indigenous fruits—at least, not in the areas where these foods have become devalued. The role of indigenous fruits in local cultures and economies varies from region to region across the world’s second-largest continent. In Kenya, for example, they were mainly eaten by young boys as they went to look after cows in the bushes and by girls when they went to look for firewood, says Mary Abukutsa, a horticultural researcher at Jomo Kenyatta University of Agriculture and Technology. Abukutsa is credited with helping to bring indigenous vegetables to popularity, and she has studied fruits to a lesser degree.

In other African countries the fruits have a market value or at least are valued by communities who have cultivated them. Katja Kehlenbeck, an ICRAF food tree scientist, says that in Sudan many indigenous fruits are still very much valued, and local inhabitants of the Nuba mountains, particularly women and children, earn a significant income from collecting and selling wild fruits.

“My general feeling is that in the more ‘traditional’ countries indigenous fruits are still valued, while in the more ‘modernized’ or ‘westernized’ countries these habits have been lost,” Kehlenbeck says. “For Kenya, there is some evidence that during colonial times the British were introducing their food and crops while putting down the value of all African foods and crops.”

According to Roger Leakey, vice chairman of the International Tree Foundation and ICRAF’s former director of research, missionaries complicated the role of these fruits in some cases by encouraging local populations to abandon the trees. “They were probably against these indigenous species as they were important in cultural activities, some of which may have been considered to be immoral,” he says. For example, some fruits were—and still are—brewed as alcoholic beverages, and others are considered to be aphrodisiacs.

But even in those areas where native fruits still have value, there is danger that it will be lost over time as well. What all indigenous fruit trees tend to have in common is that they have not been a priority for plant breeders in the past and have a lot of room for improvement. “African farmers have little if any experience with propagation techniques other than sowing seeds,” Leakey says. “There is a need for training in propagation techniques to encourage their cultivation—especially as the trees become more difficult to find in the wild.”

Farmers may see little incentive to intentionally grow indigenous fruits as a crop, because the trees are perceived as taking years to mature. This may be true in the wild, but not always when trees are cultivated, and especially when domesticated by horticultural techniques such as rooting cuttings and grafting, says Leakey. He explains that these techniques can capture the mature characteristics of the trees so that they fruit quickly, in just two to three years. Grafting can also create shorter trees with more branches, an architecture that may enable them to sequester more carbon than the normal structure of wild trees.^8^

Contrary to the idea that trees can take up a lot of space in cropping systems, Leakey says they can in fact often be cultivated in agroforestry mixtures such that the total value of all the different products produced from a plot is greater than from a single crop. And Kehlenbeck points out that the trees produce fruit for decades without much further investment.

**Figure:**
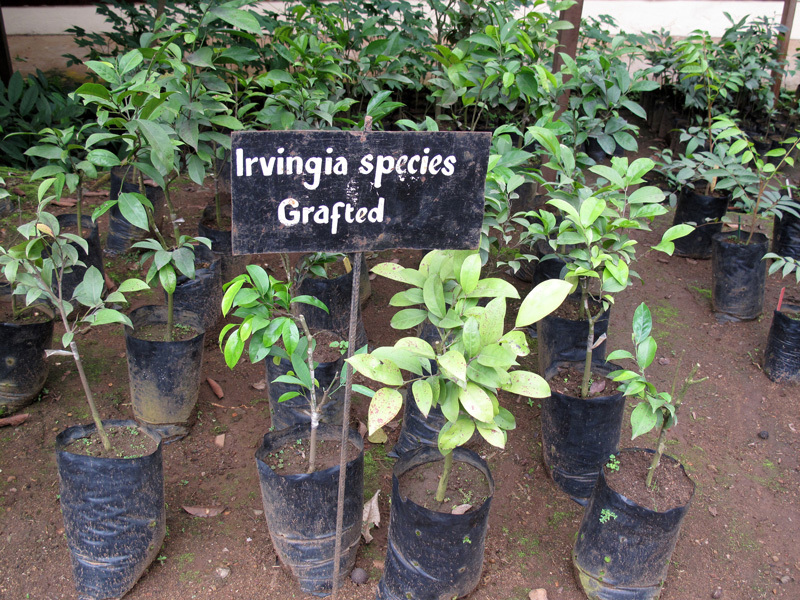
Grafted bush mango seedlings await planting. According to the World Agroforestry Centre, bush mango has been identified by farmers in Cameroon and Nigeria as their highest priority for domesticating indigenous fruit trees. © Charlie Pye-Smith/ICRAF

## Researchers and Farmers Team Up

Trees in the wild can make for difficult research subjects. They take up a lot of space, and they can grow slowly, sometimes taking decades to start bearing fruit.

Many fruit tree species have large seeds that can’t be stored for conservation or later use in a seed bank the way smaller seeds can be, according to Ian Dawson, an associate fellow at ICRAF. That’s because many of these seeds die when they are dried and cooled, processes that are necessary for seed storage. Instead, some research institutions maintain gene banks of living trees.

“People talk quite a lot about the optimum approach for tree conservation being *circa situ*,” Dawson says—that is, on farmland, close to the natural forest environment where the trees originally came from, even if the forest itself may no longer be there. Indeed, this may be the most promising approach so far to conserving indigenous fruit trees.

Across Cameroon, ICRAF researchers have been collaborating with local farmers who choose from the wild the types of trees they want to grow. Leakey says that farmers, when asked what they wanted to improve in their agricultural systems, replied that they wanted to grow the disappearing species they used to gather. ICRAF agroforesters help the farmers select and improve the best varieties, at the same time teaching them how to propagate the trees from cuttings or grafts. This process, called participatory domestication,^9^^,^^10^ started for indigenous fruit trees in Cameroon in the mid-1990s, recalls Leakey, who helped launch the project.

Today, ICRAF works in more than 500 Cameroonian villages with more than 15 types of indigenous fruit trees, including safou (*Dacryodes edulis*) and bush mango (*Irvingia gabonensis*).^11^ Ann Degrande, a socioeconomist with ICRAF in Cameroon, says some farmers plant these trees among other crops near their home, but many integrate them on larger (but still relatively small) cacao or coffee farms. Cacao and coffee are major cash crops in humid parts of West Africa, and both grow well in shade,^12^ which the fruit trees can help provide. This may be considered a form of crop intensification—assuming the cacao or coffee density is not reduced—but more importantly for ICRAF researchers, it’s also considered crop diversification “because you produce a more diverse range of [products] per unit of land,” says Degrande.

## Sowing Seeds for a Market

In some regions, businesses and organizations are trying other ways to stimulate market demand and at the same time create jobs in rural communities. Chris Dohse, managing director of Tree Crops, a Malawi-based manufacturer of wild plant ingredients, has watched a market in baobab (*Adansonia digitata*) grow almost out of nowhere.

“When we started trading [baobab products] in the second half of the 2000s, these fruits were rotting away in the forest floor, and nobody was using them,” he says. Today, he exports baobab powder to the United States and the European Union, where it is touted as a “superfood.”^13^

But the exports are dwarfed by his domestic sales. Last year, Dohse carried out an unpublished market assessment for baobab products in Malawi and calculated an annual trade of $5.5–$8.5 million. “That market didn’t exist ten years ago,” he says.

There may be a downside to this type of economic development, as Dohse is a foreigner, and foreign-run businesses don’t always provide local communities with much economic gain. But in his case, Dohse (who says he pays farmers by the kilogram) suggests that the market may not have grown in the first place without some of the marketing done by external sources.

Much of the market expansion for baobab—as well as navigation of the logistical hurdles in exporting it—can be traced to PhytoTrade Africa, a nonprofit trade association that aims to preserve indigenous biodiversity through commerce.^14^ PhytoTrade Africa members work with more than 12,000 rural producers, about 80% of whom are women. Among other projects, the Baobab Guardians Programme, run by South Africa’s EcoProducts Foundation, involves rural women who care for baobab seedlings at their homes, then plant them in the wild when they are mature enough.^15^ The guardians receive financial compensation for each completed stage of this process.

“As custodians of their surrounding forests, rural communities understand better than anyone that specific tree species cannot survive in isolation,” says Henry Johnson, market development manager for PhytoTrade Africa. “They exist as part of a wider ecosystem that must function in harmony if their target species is to flourish.”

Members are currently marketing eight indigenous fruit species—baobab, marula (*Sclerocarya birrea*), Kalahari melon (*Citrullus lanatus*), ximenia (*Ximenia* spp.), mongogo (*Schinziophyton rautanenii*), Cape mahogany (*Trichilia emetic*), kigelia (*Kigelia* spp.), and devil’s claw (*Harpagophytum* spp.). Most of these are used for cosmetics and personal care products. However, early next year, PhytoTrade Africa and Germany’s Rhine-Waal University expect to launch a project called Baofood, which will aim to promote the use, processing, and market development of baobab for improved nutritional security and rural livelihoods in Kenya and Sudan.

**Figure:**
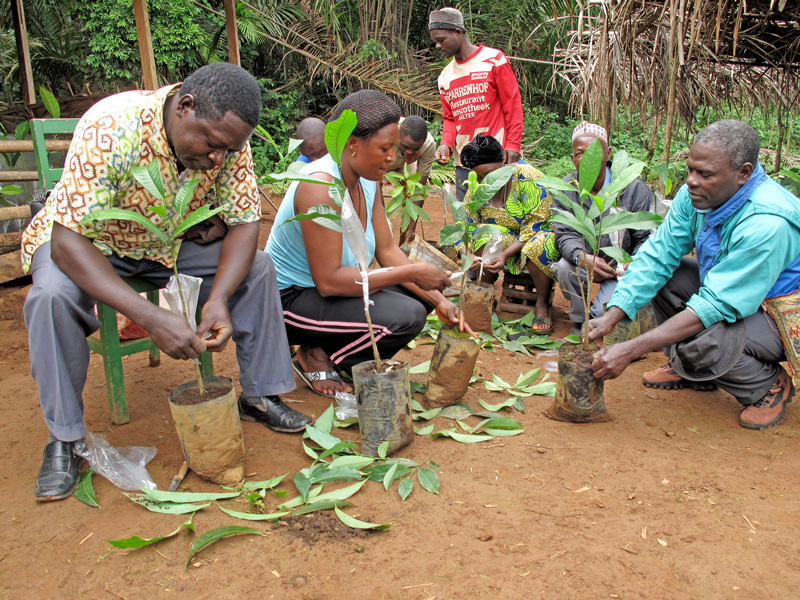
At a training session in Cameroon agroforesters train farmers in the horticultural techniques they’ll need to domesticate fruit trees. This is part of an approach known as participatory domestication. © Charlie Pye-Smith/ICRAF

## Retaining Nutritional Benefits of Indigenous Fruits

Different indigenous species produce fruit at different times throughout the year, providing nutrients to complement staple foods such as grains, roots, tubers, and pulses.^11^ A few years ago, Jamnadass and colleagues reviewed nearly 100 published papers on the nutrient contents of ten African indigenous fruits.^16^ They reported that the fruits had significant amounts of several macronutrients and micronutrients. Baobab, for example, is high in calcium, vitamin C, iron, magnesium, and zinc, while safou is a good source of magnesium, phosphorus, potassium, and zinc. Black plum (*Vitex doniana*) is rich in iron, and tamarind (*Tamarindus indica*) offers calcium, magnesium, phosphorus, and potassium.

Researchers who study domestication of Africa’s indigenous fruit trees are working in a region that already has among the highest rates of nutrient deficiencies.^17^ Therefore, they focus on preserving the trees’ nutritional value.

As agroforesters prioritize nutrition, though—or select for any particular trait—and as farmers choose specific varieties to cultivate on farms, some researchers caution that the gene pool of a tree species as a whole may be at risk of declining. This is what Dawson refers to as the paradox of domestication. “That process of selection, of domestication, by definition, results in a narrowing of the genetic base of the material,” he says. “In order to push interspecific diversity, you’re sometimes narrowing intraspecific diversity.”

But Leakey says it all depends on how domestication is done, and that ICRAF’s approach deliberately minimizes these risks. Farmers select the best specimens locally, he explains; from one village to another, or from one district to another, the development of the best trees is independent, so genetic diversity is largely maintained.

“What we’ve found is that something like seventy to eighty percent of the enormous tree-to-tree variation that exists out there in the wild also occurs at the level of a single village,” Leakey says. “If you have a hundred villages, each developing their own sets of cultivars, they’re not really narrowing the genetic base seriously. And yet they’re making this quantum leap from the wild into something that has three- to tenfold improved quality and better market potential.”

**Figure:**
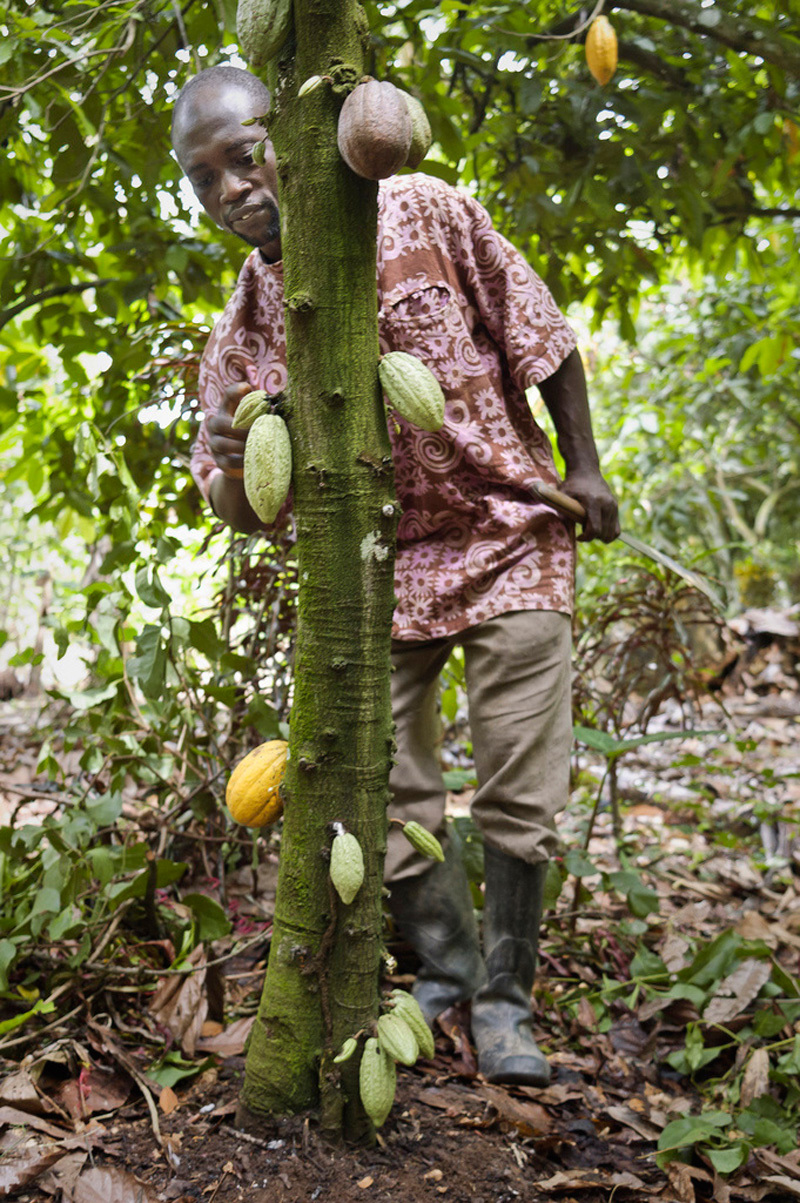
A farmer in Ghana counts the pods on a cacao tree. Many farmers are now growing indigenous fruit trees alongside major cash crops such as cacao, a practice that provides both environmental and economic benefits. © Caezer Ng

## Genome Sequencing for Trees

At the ICRAF offices in Nairobi, genetic resources unit manager Alice Muchugi has been busy at work on an entirely different aspect of indigenous fruit trees and other crops: genome sequencing. The effort is part of a broader international initiative, the African Orphan Crops Consortium,^18^ to preserve and improve so-called under-utilized crops.

Muchugi is focused on mapping the genomes of indigenous crops to create what she calls a dictionary of genetic information, and training traditional crop breeders to use this information—all with the goal of improving the crops, especially trees, in a quick and efficient way. “The genes that you are focusing on may be linked to one another. That is the information that traditional breeders don’t know—when they are selecting for a characteristic, they may be [negatively] affecting a good characteristic they had already focused on,” she says.

For example, baobab breeders may want to develop large, sweet fruit with seeds high in oil content. Knowing the tree’s genomic makeup makes it easier, Muchugi says, to breed plants that express those qualities without sacrificing others. Another potential benefit of mapping plant genomes may be the ability to identify traits that have already been bred out—and breed them back in.

Some experts question whether such advanced technologies are necessary or even valuable in the context of rural Africa, where efforts such as participatory domestication have been able to get farmers planting and caring for trees on their farms without the same expenses or training. Muchugi and other proponents say that with the relatively slow speed of domestication, particularly for trees, it saves invaluable amounts of time, measurable in years.

Jamnadass, who oversees a team that is working with both strategies, thinks each approach has a role to play. “We’ve got the downstream [i.e., participatory] approaches, and now with the genomics, we’ve got the upstream approaches,” she says. “We feel like we’re in a really good place, bridging these two approaches to come up with the best varieties and cultivars, and at least provide the best training to those who will develop their crops,” she says.

For Leakey, scaling up cultivation of indigenous fruit trees to truly reverse their decline will depend on decision makers in agricultural development. He says, “The idea of working with traditional crops is something that many people may consider a luxury they would do if they had a little corner of their garden. What I’m saying is that actually this is not a luxury; this is an absolute necessity for the future of tropical agriculture.”

**Figure:**
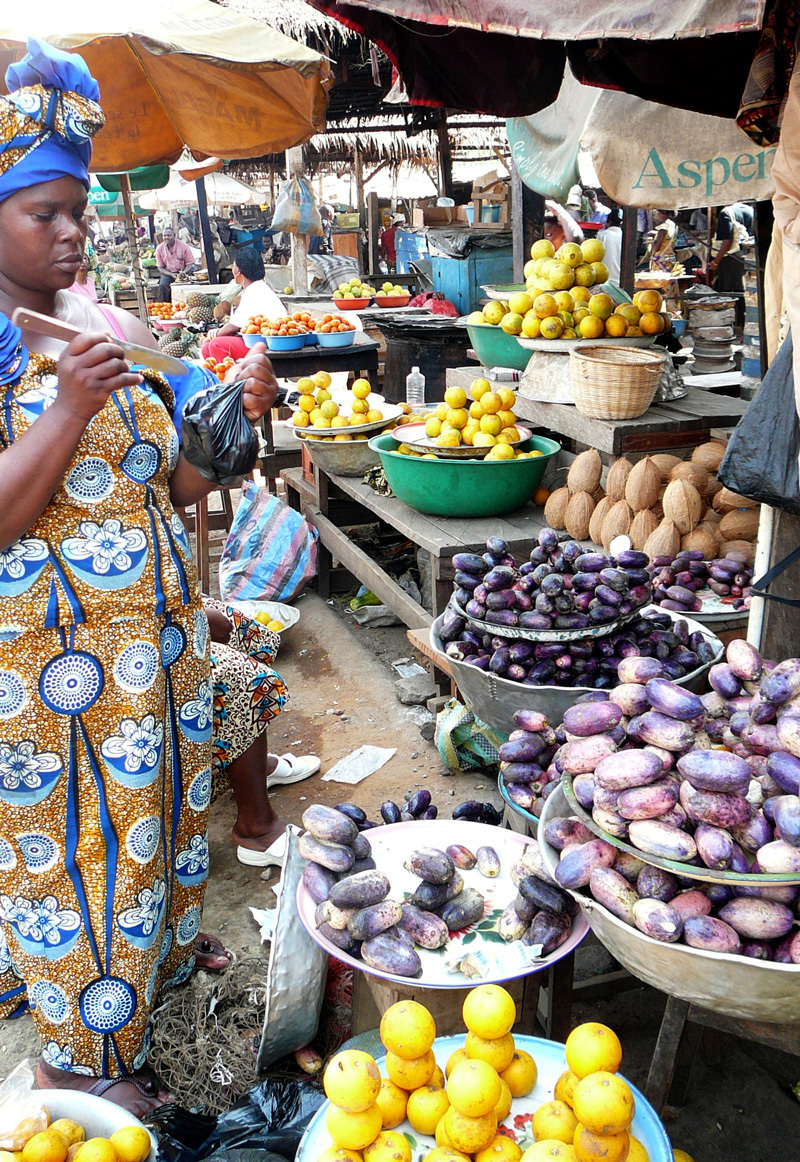
In African countries with less of a western influence, many people still value indigenous fruits such as the purple safou sold in this market. But even in these countries, farmers need training to help them ensure a future for native fruit trees, which are rapidly disappearing from the wild. © Roger Leakey
